# Particulate matter-attributable mortality and relationships with carbon dioxide in 250 urban areas worldwide

**DOI:** 10.1038/s41598-019-48057-9

**Published:** 2019-08-09

**Authors:** Susan C. Anenberg, Pattanun Achakulwisut, Michael Brauer, Daniel Moran, Joshua S. Apte, Daven K. Henze

**Affiliations:** 1 Milken Institute School of Public Health, George Washington University, Washington DC, USA; 2 School of Population and Public Health, University of British Columbia, Vancouver, British Columbia Canada; 3 Institute for Health Metrics and Evaluation, University of Washington, Seattle, Washington USA; 4 Norwegian University of Science and Technology, Trondheim, Norway; 5 Department of Civil, Architectural and Environmental Engineering, University of Texas – Austin, Austin, Texas USA; 6 University of Colorado – Boulder, Boulder, Colorado USA; 7 Stockholm Environment Institute, Seattle Washington, USA

**Keywords:** Risk factors, Environmental impact

## Abstract

Urban air pollution is high on global health and sustainability agendas, but information is limited on associated city-level disease burdens. We estimated fine particulate matter (PM_2.5_) mortality in the 250 most populous cities worldwide using PM_2.5_ concentrations, population, disease rates, and concentration-response relationships from the Global Burden of Disease 2016 Study. Only 8% of these cities had population-weighted mean concentrations below the World Health Organization guideline for annual average PM_2.5_. City-level PM_2.5_-attributable mortality rates ranged from 13–125 deaths per 100,000 people. PM_2.5_ mortality rates and carbon dioxide (CO_2_) emission rates were weakly positively correlated, with regional influences apparent from clustering of cities within each region. Across 82 cities globally, PM_2.5_ concentrations and mortality rates were negatively associated with city gross domestic product (GDP) per capita, but we found no relationship between GDP per capita and CO_2_ emissions rates. While results provide only a cross-sectional snapshot of cities worldwide, they point to opportunities for cities to realize climate, air quality, and health co-benefits through low-carbon development. Future work should examine drivers of the relationships (e.g. development stage, fuel mix for electricity generation and transportation, sector-specific PM_2.5_ and CO_2_ emissions) uncovered here and explore uncertainties to test the robustness of our conclusions.

## Introduction

Urban air pollution is high on the global sustainable development agenda^1–3^. The world’s urban population is expected to grow from >50% of today’s global population to 66% by 2050^4^, with urban areas projected to absorb all population growth. Efforts to address urban air pollution by intergovernmental organizations, global networks (e.g. C40 cities, Global Urban Air Pollution Observatory), national governments, and individual cities can benefit from quantitative estimates of urban air pollution-related health impacts. Such estimates can help prioritize mitigation actions in cities (e.g. investing in electric buses, public transportation, and active urban mobility) and can motivate national scale policies (e.g. ambient air quality standards, emission standards for sources such as vehicles). Furthermore, since combustion is a major source of greenhouse gases and air pollution^5^, cities can reap immediate and local health benefits while also contributing to reductions of combustion-related climate-forcing pollutants^6^. Air pollution disease burdens by source sector have been quantified at the national level^7,8^ and city level for individual cities^9–11^ but information is limited for cities globally.

Ambient PM_2.5_ is considered the leading environmental health risk factor globally and is a top 10 risk factor in countries across the economic development spectrum^12^. Early studies estimating the global burden of disease from air pollution focused on cities, where most of the world’s ground-based monitors were located^13^. Currently the most comprehensive global burden of disease studies report estimates at the national scale (sub-national for some countries)^12,14^, enabled by the full global coverage and high resolution of satellite remote sensing of aerosol optical depth^15^. Here, we exploit these global, highly resolved PM_2.5_ concentrations to estimate the burden of disease attributable to PM_2.5_ in 250 major cities worldwide. Unlike previous estimates of air pollution disease burdens among subsets of cities^16–18^, our globally consistent methods enable comparisons across cities worldwide and are compatible with the Global Burden of Disease 2016 (GBD 2016) Study^12^.

## Results

**Figure 1:**
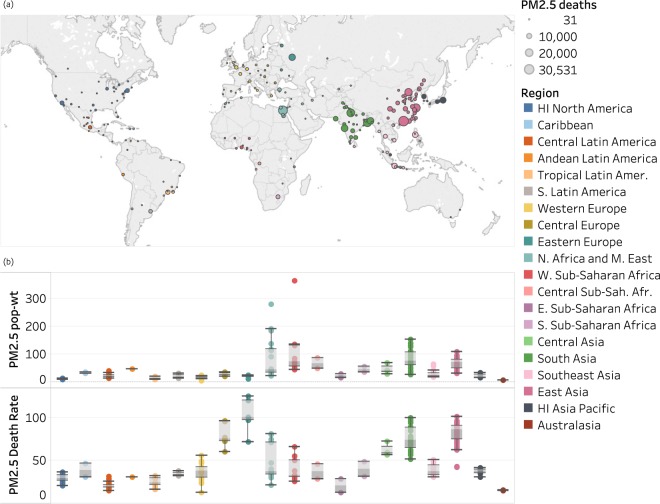
PM_2.5_-attributable premature deaths in 2016 in 250 cities worldwide. (**a**) Number of PM_2.5_-attributable deaths on a world map; (**b**) Box plots of population-weighted annual average PM_2.5_ concentration (PM2.5 pop-wt) and PM_2.5_ attributable deaths per 100,000 people (PM2.5 death rate) across all cities in each region. Boxes indicate the middle 50% of the data; whiskers show data within 1.5 times the interquartile range. HI = High-Income.

We first estimated PM_2.5_-attributable mortality in 2016 for the 250 most populous urban areas (see Methods regarding the city definition). The median population-weighted PM_2.5_ concentration was 29 µg/m^3^ [standard deviation (sd) = 43 µg/m^3^, range 5–365 µg/m^3^; Fig. 1], three times greater than the WHO guideline for annual average PM_2.5_ (10 µg/m^3^). Among the 250 cities, only 21 (8%, all in Sweden, the US, Canada, Australia, and Brazil) had population-weighted mean concentrations below the guideline, whereas 104 (42%) exceeded the WHO Interim Target 1 (35 µg/m^3^). The median rate of PM_2.5_-attributable deaths was 39 deaths per 100,000 people (sd = 26, range 13–125 per 100,000 people; Fig. 1). Several regions show large variability in city-specific rate of PM_2.5_-attributable deaths (Fig. 1). While the top 10 cities for population-weighted PM_2.5_ were mostly in Africa and Asia, the top 10 for PM_2.5_-attributable mortality rate were all in Asia and Europe (Fig. S1 and Table S1), driven by high cardiopulmonary disease rates in Europe and high PM_2.5_ concentrations in Asia. High concentrations in Northern Africa and Middle East cities are partly driven by wind-blown mineral dust, which is mostly naturally-occurring. Cities in Australia, Brazil, Canada, Sweden, and the U.S. that had PM_2.5_ concentrations below the WHO guideline were in the lowest quartile of PM_2.5_-attributable mortality rates among these 250 cities.

**Figure 2:**
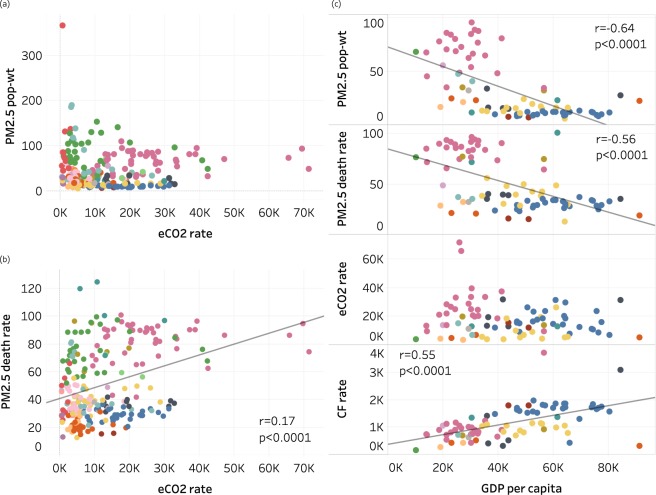
City-specific estimates of PM_2.5_-attributable premature deaths per capita in 2016 versus other city indicators. (**a**) Population-weighted annual average PM_2.5_ concentration (µg/m^3^) vs. annual CO_2_ emissions rate (t C per 100,000 people); (**b**) PM_2.5_ death rate (deaths per 100,000 people) vs. annual CO_2_ emissions rate; (**c**) comparison of population-weighted PM_2.5_, PM_2.5_ death rate, CO_2_ emissions rate, and 2013 carbon footprint rate (kt CO_2_ per 100,000 people) vs. GDP per capita ($) in 2015 in 82 cities. Colors indicate world regions (see Fig. 1 legend). Linear regression lines are shown where correlations are significant, r is the correlation coefficient, and p is the correlation significance level. (Note: Riyadh was removed from panels a and b to show more detail in the rest of the dataset. Its CO_2_ emission rate is likely unrealistically high due to very low population estimate in the GPWv4 dataset: CO_2_ emission rate = 290,000 kt CO_2_ per 100,000 people, PM_2.5_ pop-wt = 280 µg/m^3^, and PM_2.5_ death rate = 40.) Similar graphs for each region (using “super-regions” from the Global Burden of Disease 2016 Study) and the 50 most populous cities globally are in the Supplemental Information (Figs S5–S12).

To explore whether cities with high particulate air pollution are also large CO_2_ emitters, we compared city-level PM_2.5_ concentrations and mortality rates to local CO_2_ emissions. We found no association between PM_2.5_ concentrations and CO_2_ emission rates (Fig. 2a). PM_2.5_ mortality rates and CO_2_ emission rates were weakly positively correlated, though with regional influences on PM_2.5_ mortality rates apparent from clustering of cities in the same region (Fig. 2b and Fig. S2). This clustering may result from national-scale policies, regional pollution transport, and other factors (e.g. geographical or meteorological) affecting many cities simultaneously. The national disease rates used in this study also contribute to regional clustering in the PM_2.5_ death rates. Many Asian cities are among the highest for PM_2.5_ mortality rate but only 10 Asian cities emit more CO_2_ per 100,000 people than the largest high-income emitters. Contrastingly, high-income North American cities have low PM_2.5_ mortality rates but mid- to-high CO_2_ emissions rates. European and African cities range from low to very high for PM_2.5_ mortality rates but African cities are relatively low and European cities in the mid-range for CO_2_ emissions rates. To explore the influence of economic development, we compared population-weighted PM_2.5_ concentration, PM_2.5_-attributable mortality rates, and CO_2_ emissions to city-level gross domestic product (GDP; Fig. 2c). Across 82 cities with available city-specific GDP data, PM_2.5_ concentrations and mortality rates were negatively associated with city GDP per capita, but no relationship exists between GDP per capita and CO_2_ emissions rates.

To further elucidate why PM_2.5_ concentrations and mortality decline more than CO_2_ emissions with increasing GDP, we compared PM_2.5_ deaths against consumption-based carbon footprints, which account for CO_2_ emitted worldwide from production of locally-consumed goods. North American and European cities, which are high consumers of products manufactured elsewhere, are ranked higher among the 250 cities for carbon footprints compared with local CO_2_ emissions (Fig. S3). The opposite is true for most Asian cities, where export-dominated manufacturing prevails. The positive relationship between GDP per capita and carbon footprint is expected since GDP was an input to estimate urban carbon footprints^19^. The pattern of large carbon footprints but low PM_2.5_ mortality rates in North American cities, and small carbon footprints but high PM_2.5_ mortality rates in Asian cities potentially indicates that many cities with large carbon footprints (e.g. U.S. cities) have exported PM_2.5_ and health impacts to other places (e.g. Asian cities) which manufacture consumption goods that are then imported elsewhere, as explored previously e.g. ^20^. To identify cities that are performing better or worse than predicted by the linear per capita GDP-PM_2.5_ deaths relationship, we examined the regression residuals. Mexico City, Monterrey, Rio de Janeiro, Sao Paolo and Melbourne had lower PM_2.5_ mortality rates compared with their predicted values, while Wuxi, Tianjin, Wuhan, Moscow and Warsaw had higher mortality rates than expected based upon GDP per capita.

## Discussion

These analyses provide the first estimates of the PM_2.5_ disease burden in urban areas worldwide using methods that are globally consistent (enabling comparisons across cities globally) and compatible with the Global Burden of Disease 2016 Study. Estimated PM_2.5_-attributable deaths per 100,000 people varied by a factor of 10 across the 250 most populous cities worldwide, indicating that some cities are achieving far lower levels of air pollution-related health impacts than others. We found a weakly positive correlation between PM_2.5_ mortality and CO_2_ emission rates, which suggests that there may be opportunities for cities to achieve climate and air quality co-benefits through mitigation measures that address both PM_2.5_ and CO_2_. In contrast, we found that while regions with wealthier cities have reduced their PM_2.5_ concentrations and mortality burdens considerably, CO_2_ emissions have not declined in parallel. This first cross-sectional snapshot of cities globally does not allow for drawing strong conclusions as to the factors driving these relationships. However, we suspect that several explanations for these relationships may be occurring in concert: (1) historical tendency in developed countries to address air quality by implementing end-of-pipe emission controls that reduce air pollution but not carbon (e.g. diesel particulate filters on vehicles, scrubbers that remove sulfur dioxide emissions from power plants); (2) movement of industry and power generation out of cities, while the relatively “clean” energy sources remaining in cities still produce CO_2_ emissions; (3) “out-sourcing” manufacturing and associated pollution from wealthy cities to other locations around the world, where lax environmental regulations may result in more emissions per unit energy consumed. While the first factor reduces PM_2.5_ levels, the second two simply move pollution from one place to another without necessarily improving air quality overall. Future research could examine these and other characteristics of cities, such as development stage, fuel mix for electricity generation and transportation, and sector-specific emissions of PM_2.5_ and CO_2_, in more detail and over time, to further elucidate the drivers of the relationships uncovered here.

The world faces a challenge as urbanization rapidly expands populations mainly in Asian and African cities, where PM_2.5_ levels are also mostly trending upward^21^. This initial analysis of city air pollution burdens using globally consistent methods paints a salient yet still emerging lesson: to slow climate change, improve air quality, and protect public health simultaneously, historically “successful” air quality management programs may not be enough. Low carbon development, however, can avoid the fossil fuel combustion that releases both air pollution and greenhouse gases. As air pollution remains a top 10 risk factor for most countries globally, all cities, even those with relatively low PM_2.5_ mortality rates, can improve local public health by transitioning away from fossil fuels. Thus, the challenge of urban PM_2.5_ can also be viewed as an opportunity – reducing fossil fuel combustion offers local and immediate air quality and public health benefits, in addition to slowing climate change globally and over centuries. This opportunity can be realized in many ways, including by improving building energy efficiency, displacing vehicular traffic with active transportation, electrifying public transportation, and transitioning to renewables for power generation. Several of these approaches would have additional co-benefits from fewer road traffic collisions, more physical activity, less noise pollution, and other improvements.

Several limitations may affect the strength of our conclusions. While our top-down, globally consistent approach offers consistency and broad coverage (providing PM_2.5_ mortality estimates for many cities which otherwise would have none), bottom-up and local data could improve estimates for individual cities. For example, though we used national disease rates, subnational disease rates can vary by ±20–40% or more compared to national average rates^22^. This additional heterogeneity is not captured here, but is small relative to the global differences we estimate. We neglected uncertainty in the input variables, though PM_2.5_ concentrations, relative risks, CO_2_ emissions, carbon footprints, and city GDP are each uncertain and may vary between existing datasets and inventories^23^. PM_2.5_ concentrations are uncertain because much of the world still lacks ground monitoring networks, though most monitors included by Shaddick *et al*.^15^ were in cities. Beyond PM_2.5_, urban populations are also exposed to ground-level ozone, nitrogen dioxide, and other combustion-related air pollutants. PM_2.5_ is also associated with other health outcomes, including asthma^24^, excluded here for consistency with the 2016 GBD. Our analysis is cross-sectional and could be supplemented with future longitudinal analysis to identify determinants of PM_2.5_-CO_2_ relationships (e.g. city size, population, and geographical location) and consider other climate warming pollutants. Exploring uncertainties and their influences on city-level PM_2.5_-attributable mortality estimates could also test the robustness of these results and conclusions.

## Methods

We estimated PM_2.5_ health impacts using PM_2.5_ concentration (0.1° × 0.1° grid resolution)^15^, population, national baseline disease rates, and concentration-response relationships from the GBD 2016^12,25^. Annual average PM_2.5_ concentrations were estimated by combining satellite-derived aerosol optical depth with vertical aerosol distribution from a chemical transport model, calibrated to 6,003 measurements from 117 countries. Gridcell concentrations ranged from 0.9 to 990 μg/m^3^ globally. Gridded population counts aggregated to 0.1° × 0.1° are from the CIESIN Gridded Population of the World v4 (total in 2016 was 7.28 billion; http://sedac.ciesin.columbia.edu/data/collection/gpw-v4, accessed August 17, 2018). We downloaded country-, age-, and cause- specific baseline deaths in 2016 from the GBD Data Exchange (http://ghdx.healthdata.org/gbd-results-tool, accessed June 1, 2018).

We calculated age- and cause-specific relative risk of disease for each gridcell PM_2.5_ concentration using Integrated Exposure Response (IER) curves^25^. The shape of the IERs depends on the health endpoint, and flattens at very high concentrations, particularly for cardiovascular endpoints. We created lookup tables in 0.1 µg/m^3^ increments of PM_2.5_ concentration, following previous studies^26,27^. Central estimates of PM_2.5_-attributable health impacts were calculated using the mean of the 1000 IER parameter draws for each health endpoint, and 95% confidence intervals were calculated using the 2.5^th^ and 97.5^th^ percentiles. We applied theoretical minimum risk exposure levels included with the IER parameter dataset from a uniform distribution of 2.4 to 5.9 µg/m^3^. All calculations were performed in MATLAB r2013b and R v3.4.2.

Globally, we estimate that ambient annual average PM_2.5_ in 2016 was associated with 4.1 million deaths (95% confidence interval, 2.3–6.1 million), within 0.3% of GBD 2016 results^25^. Approximately 20%, 39%, 19%, 7%, and 16% were from stroke, ischemic heart disease, chronic obstructive pulmonary disease, lung cancer, and lower respiratory infections, respectively.

For city-specific PM_2.5_ mortality, we summed gridded PM_2.5_ mortality estimates within urban spatial extents from the Global Human Settlement grid (GHS-SMOD) for 2015 at 1 km resolution (https://ghsl.jrc.ec.europa.eu/ghs_smod.php, Accessed August 17, 2018)^28^. We defined cities following the “urban centers or high density clusters” definition, with ≥1,500 inhabitants per km^2^ or a density of built-up ≥50% and ≥50,000 inhabitants. We matched GHS-SMOD city identifiers to city names in ArcGIS. GHS-SMOD city definitions treat patches of dense contiguous urban fabric (e.g. Tokyo-Kawasaki-Kawagoe-Hachioji-Yokohama) as one large “city”. Scaling the 1 km urban definition grid to the 0.1° × 0.1° resolution of our disease burden estimates resulted in loss of urban spatial extent, population, and air pollution-attributable deaths compared with the finer resolution. Therefore, to retain as much data as possible, we multiplied our estimated air pollution-attributable deaths in each urban area at 0.1° × 0.1° by the ratio of population in each urban area calculated at high-resolution (0.0083° × 0.0083°, or ~1 km) versus low resolution (0.1° × 0.1°).

City fossil fuel CO_2_ emissions in 2016 are from the Open-source Data Inventory for Anthropogenic CO_2_ (ODIAC), a globally gridded (1 km) satellite-derived dataset^29^. City carbon footprints (for 2013) are from recently published estimates for 13,000 cities using the same GHS-SMOD city definitions (http://citycarbonfootprints.info/, Accessed August 17, 2018)^19^. Briefly, national carbon footprints were spatially allocated based on population, purchasing power, and existing subnational estimates from the U.S., China, the European Union, and Japan. CO_2_ emissions are production-based, while carbon footprints are consumption-based. GDP estimates for 2015 are from a Brookings Institution report^30^. Statistical associations are indicated for a significance level of p < 0.05.

Population-normalized rates were calculated using the GBD population dataset used to calculate PM_2.5_ mortality, except carbon footprints which were estimated with GHS-POP population. Fig. S4 compares the two population datasets.

## Supplementary information

Supplemental Material

Supplemental Data

## Data Availability

Results for all 250 urban areas, including cities within each urban cluster, country, region, PM_2.5_ concentrations, and PM_2.5_ mortality are available at: https://figshare.com/articles/_/7871747. All other data used in this study are either publicly available or are available from the authors upon request.
