# Supplementary material for: Particulate matter-attributable mortality and relationships with carbon dioxide in 250 urban areas worldwide
Source: Sci Rep. 2019 Aug 9;9:11552. doi: 10.1038/s41598-019-48057-9 (PMC6689059; doi:10.1038/s41598-019-48057-9)
Supplement: Supplementary file 1 — Supplemental Material [file 41598_2019_48057_MOESM1_ESM.docx]

**Particulate matter-attributable mortality and relationships with carbon dioxide in 250 urban areas worldwide**

Susan C. Anenberg, Pattanun Achakulwisut, Michael Brauer, Daniel Moran, Joshua S. Apte, Daven K. Henze

**Supplemental Information**

This supplemental material contains additional figures and tables. City-specific results for population-weighted PM_2.5_ concentrations, PM_2.5_ deaths by cause, CO_2_ emissions, and carbon footprints are provided in a supplemental appendix.

**Supplemental Figures**


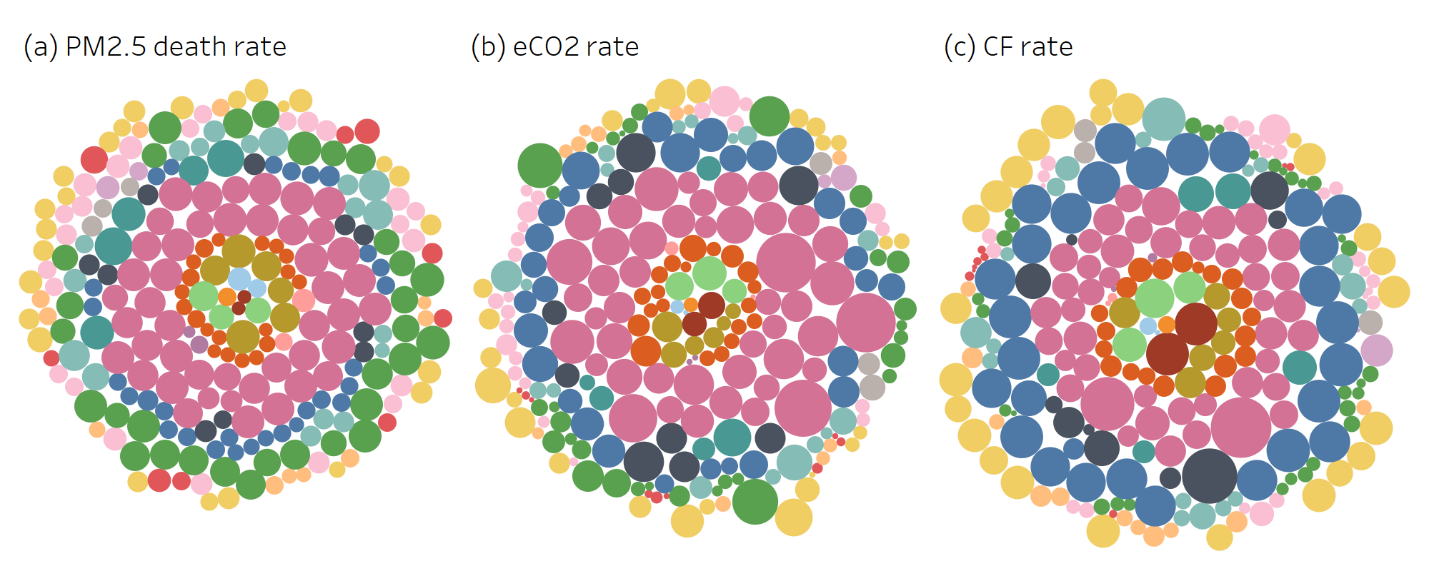


Figure S 1. City-specific estimates in 2016 for: (a) PM_2.5_-attributable deaths per 100,000 people; (c) CO_2_ emissions rate (t C per 100,000 people); (d) carbon footprint rate (kt CO_2_ per 100,000 people). Bubbles are sized according to the city-specific value in each plot. Bubbles are generally grouped by region but position is random. Riyadh (CO_2_ emission rate = 290,000 kt CO_2_ per 100,000 people) was removed from panel b to show more detail in the rest of the dataset (CO_2_ emission rate is likely unrealistically high due to very low population estimate in the GPWv4 dataset). Colors represent world regions – see Figure 1 for color legend.


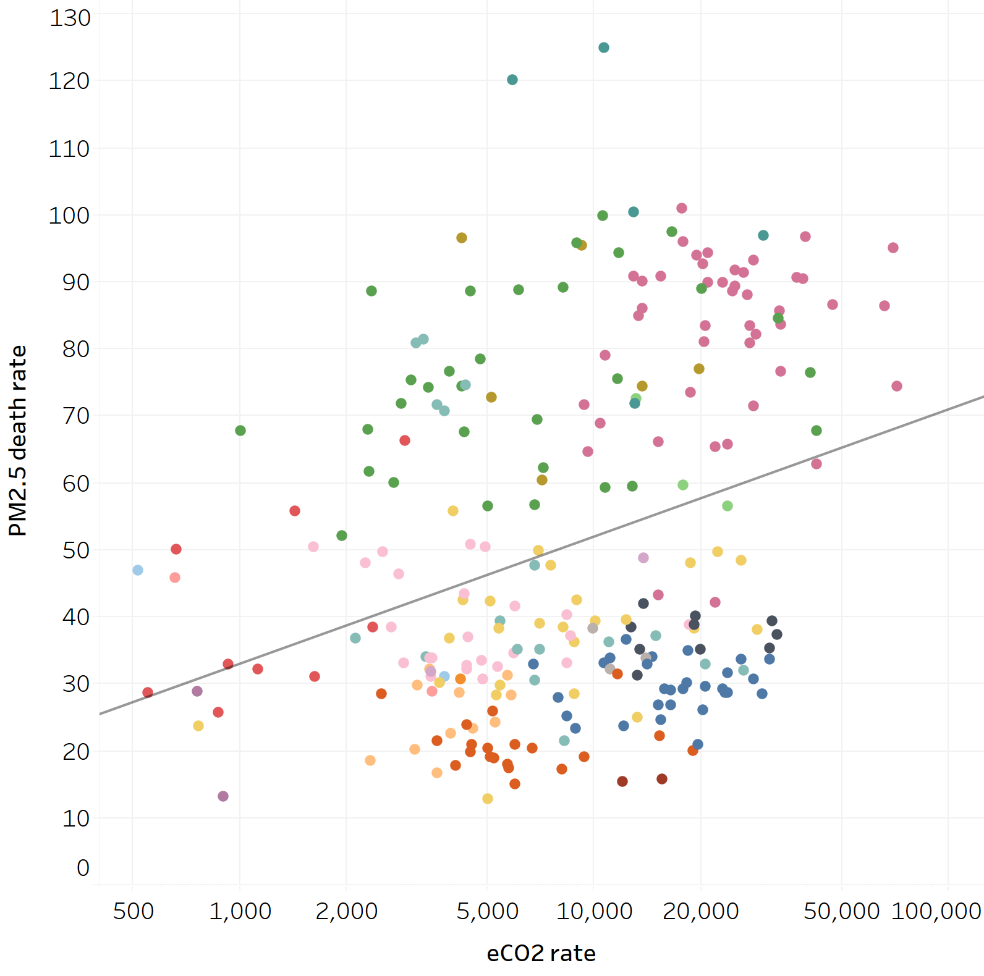


Figure S 2. As for Figure 2b but with CO_2_ emissions rate on a log scale to show data points at the low end of the scale more clearly. The trendline depicted here shows a log-linear relationship. See Figure 1 for color legend.


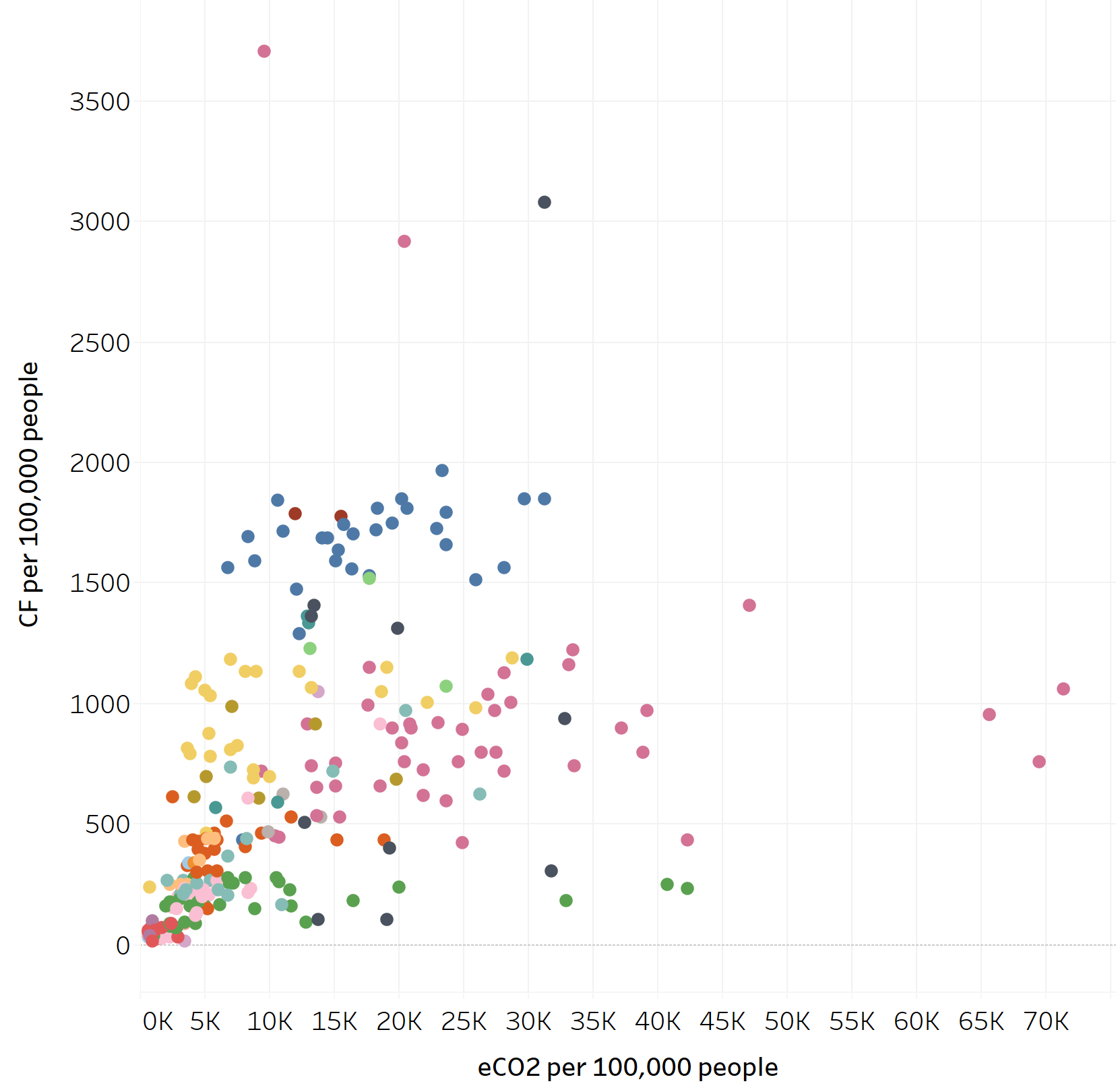


Figure S 3. Carbon footprint rate (kt CO_2_ per 100,000 people per year) vs. CO_2_ emissions rate (t C per 100,000 people per year) for each city. Riyadh (CO_2_ emission rate = 290,000 kt CO_2_ per 100,000 people and carbon footprint rate= 1900 t CO_2_ per 100,000 people) was removed to show more detail in the rest of the dataset (CO_2_ emission rate is likely unrealistically high due to very low population estimate in the GPWv4 dataset). See Figure 1 for color legend.


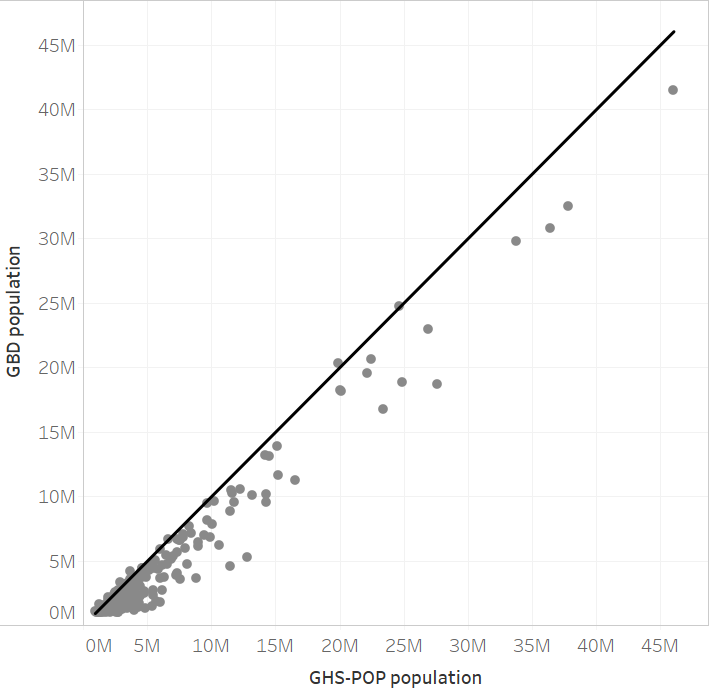


Figure S 4. Comparison between 2016 population from the GBD 2016 dataset versus GHS-POP in each city. Black line indicates 1:1 line.


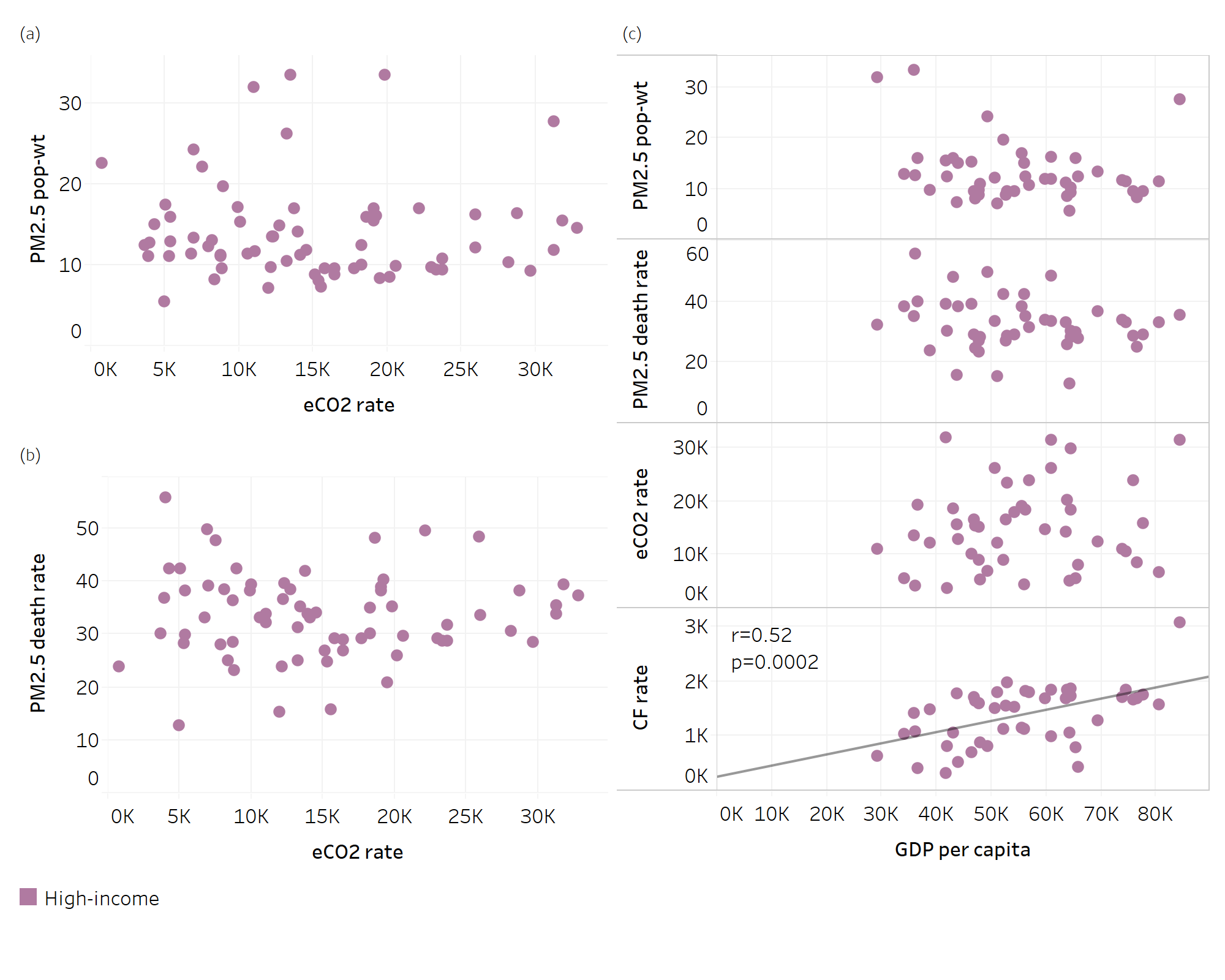


Figure S 5. As for Figure 2, but for the High-income super-region (n=69 for panels a and b, n=46 for panel c).


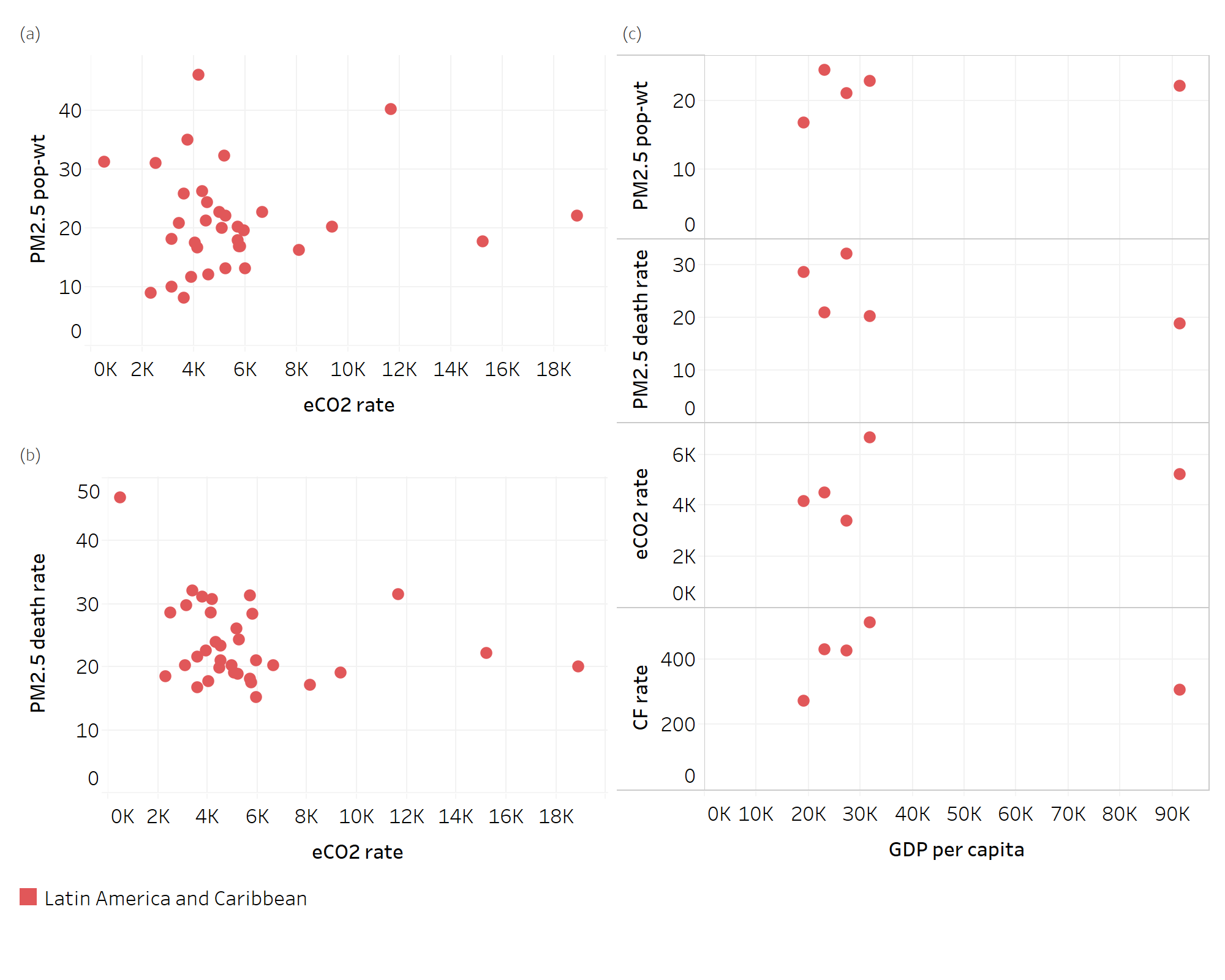


Figure S 6. As for Figure 2, but for the Latin America and Caribbean super-region (n=34 for panels a and b, n=5 for panel c).


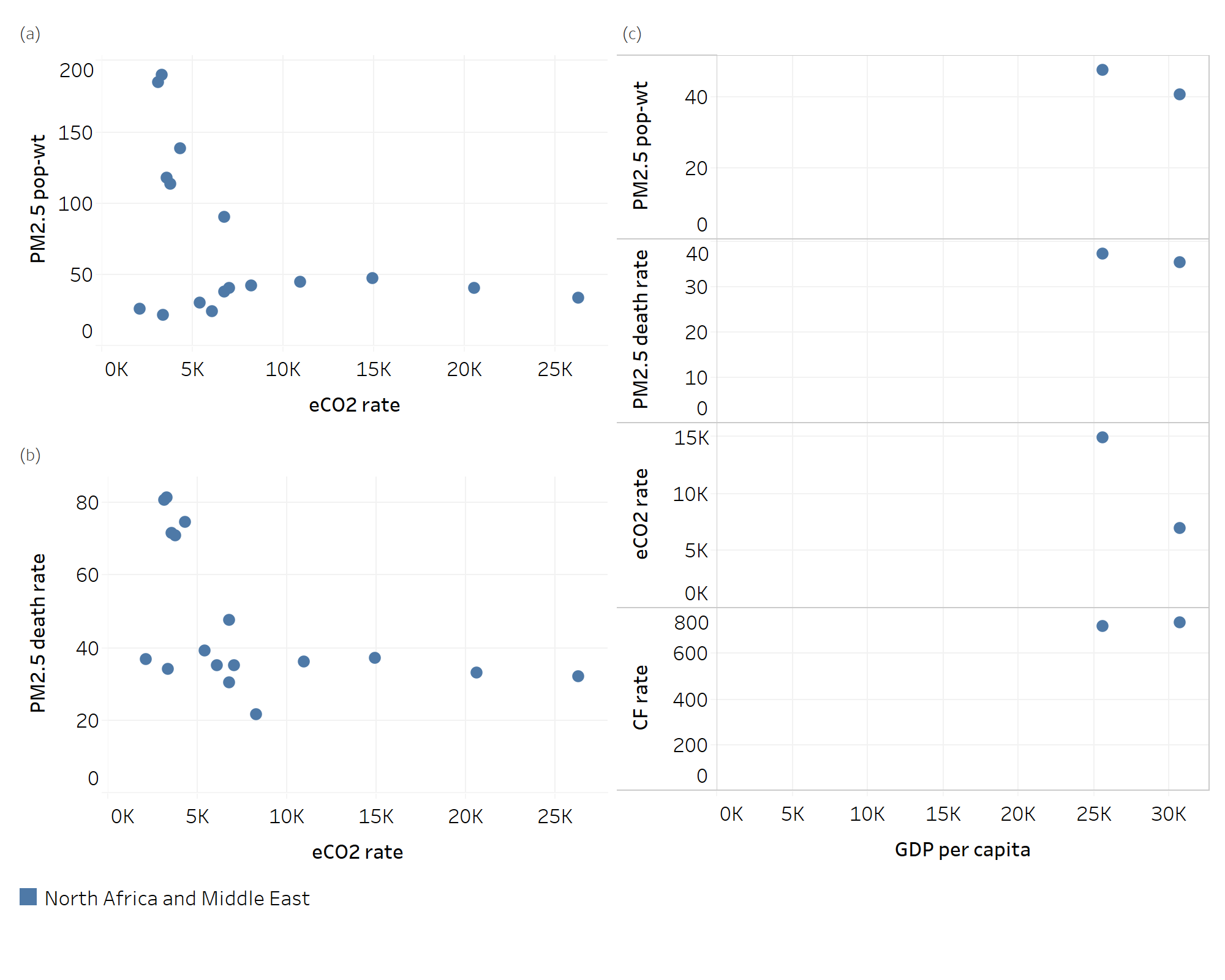


Figure S 7. As for Figure 2, but for the North Africa and Middle East super-region (n=18 for panels a and b, n=2 for panel c).


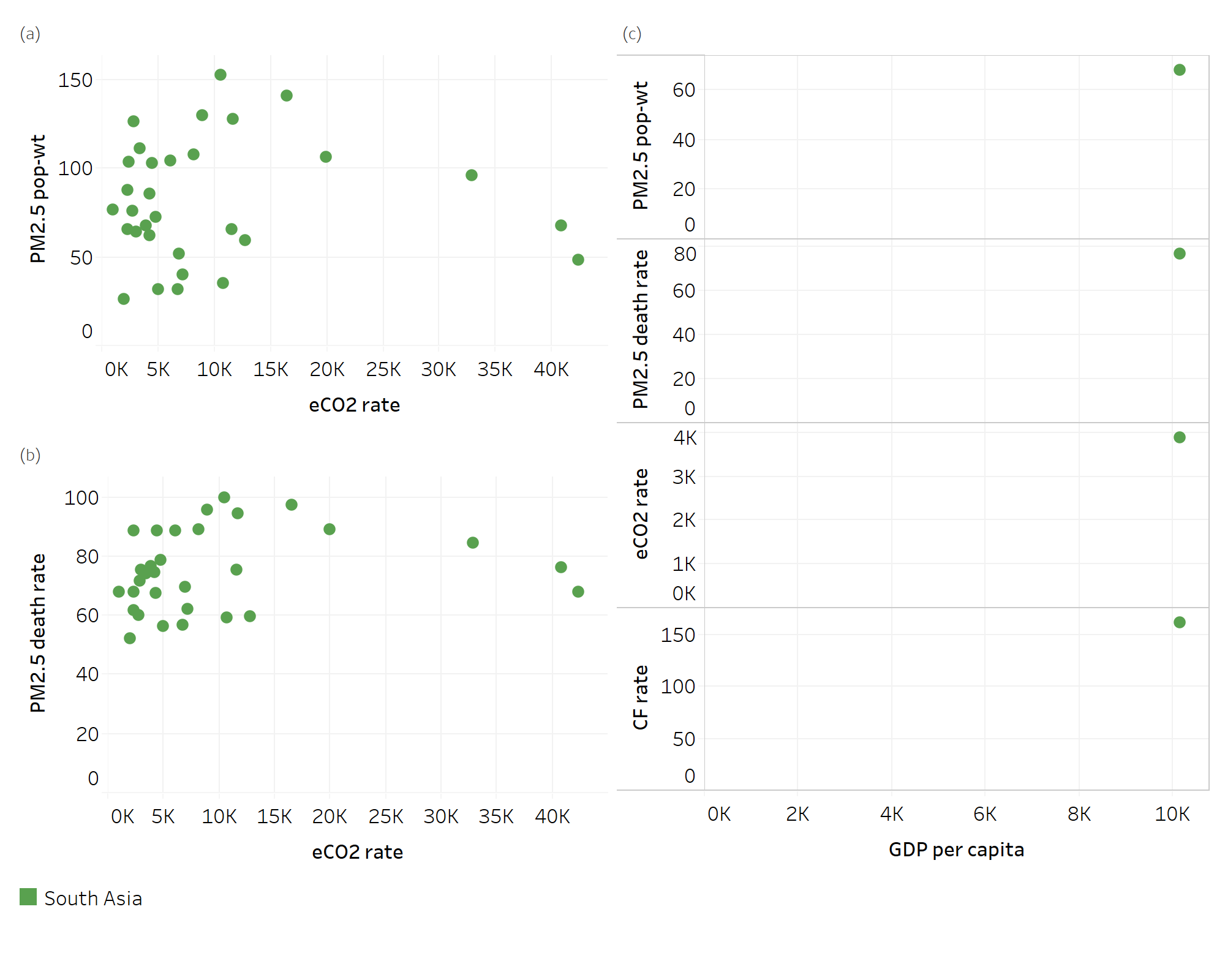


Figure S 8. As for Figure 2, but for the South Asia super-region (n=31 for panels a and b, n=1 for panel c).


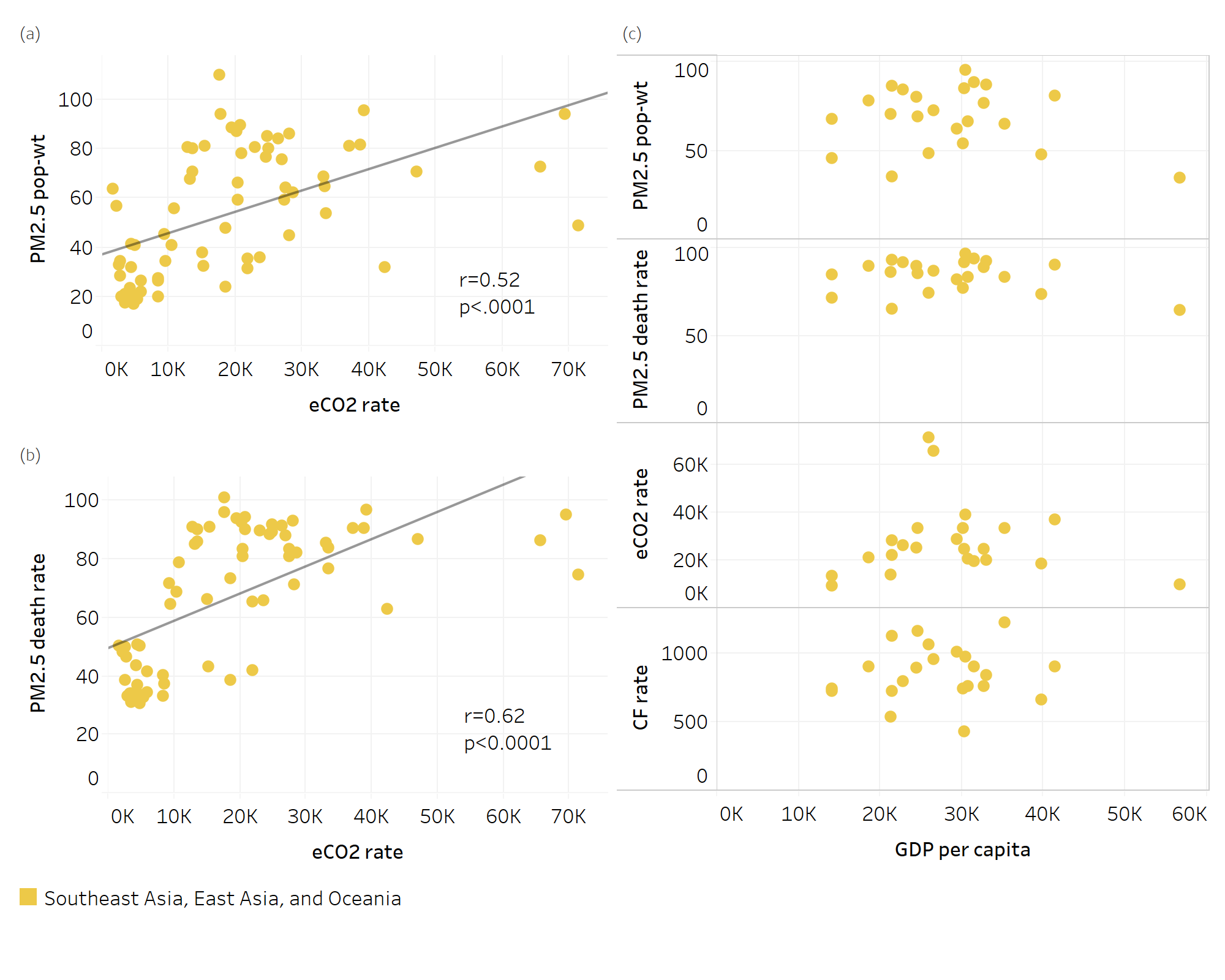


Figure S 9. As for Figure 2, but for the Southeast, East Asia, and Oceania super-region (n=69 for panels a and b, n=23 for panel c).


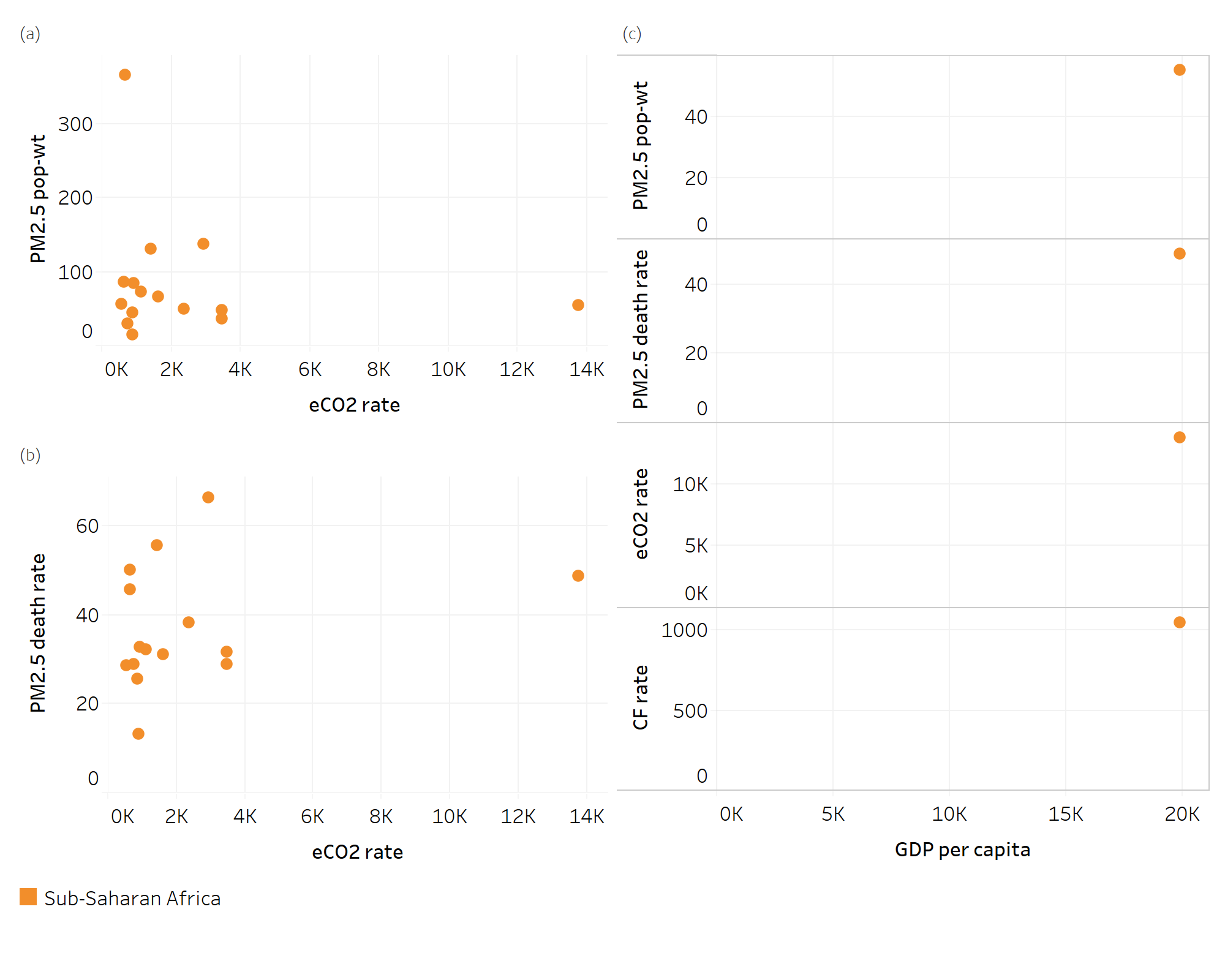


Figure S 10. As for Figure 2, but for the Sub-Saharan Africa super-region (n=15 for panels a and b, n=1 for panel c).


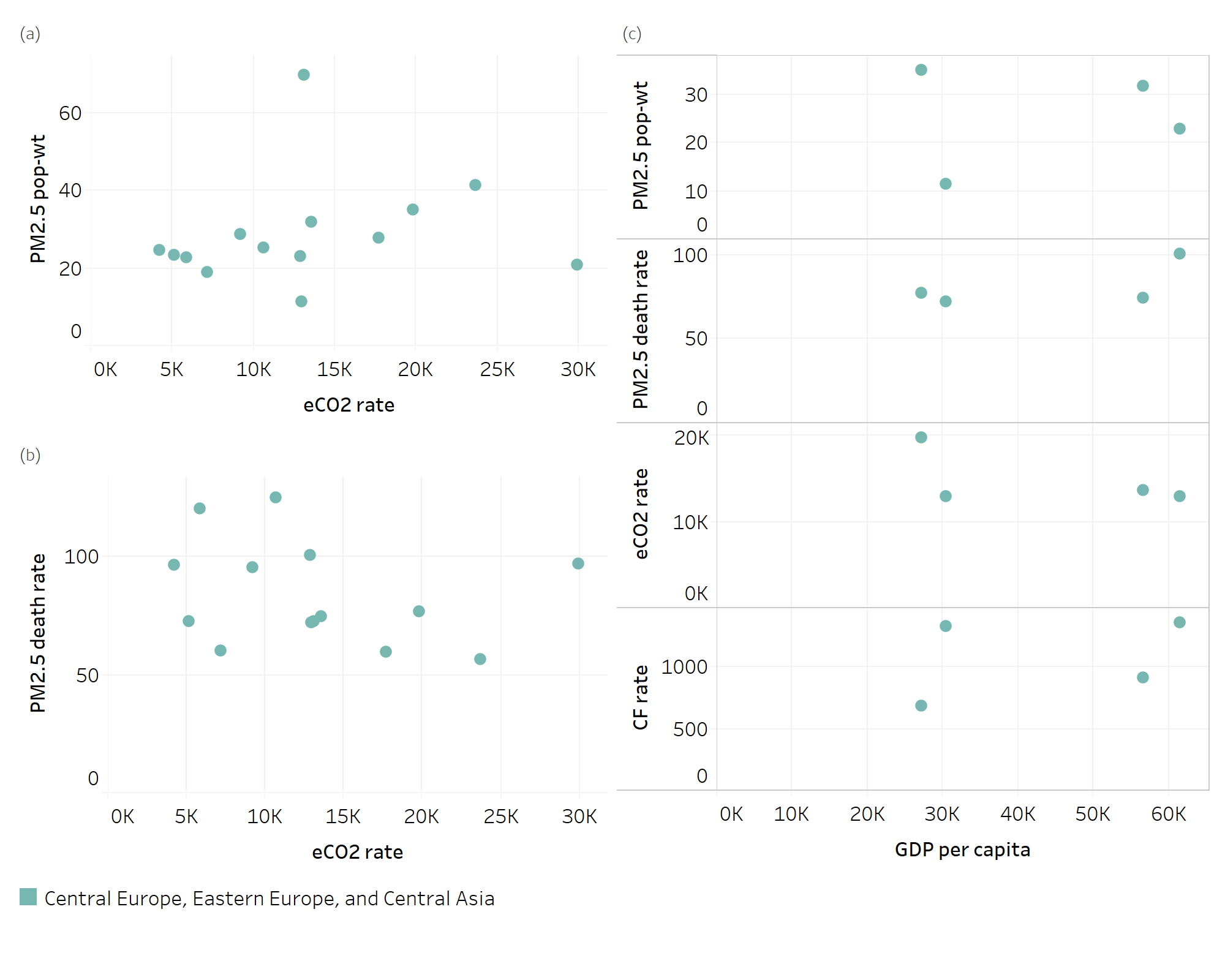


Figure S 11. As for Figure 2, but for the Central Europe, Eastern Europe, and Central Asia super-region (n=14 for panels a and b, n=4 for panel c).


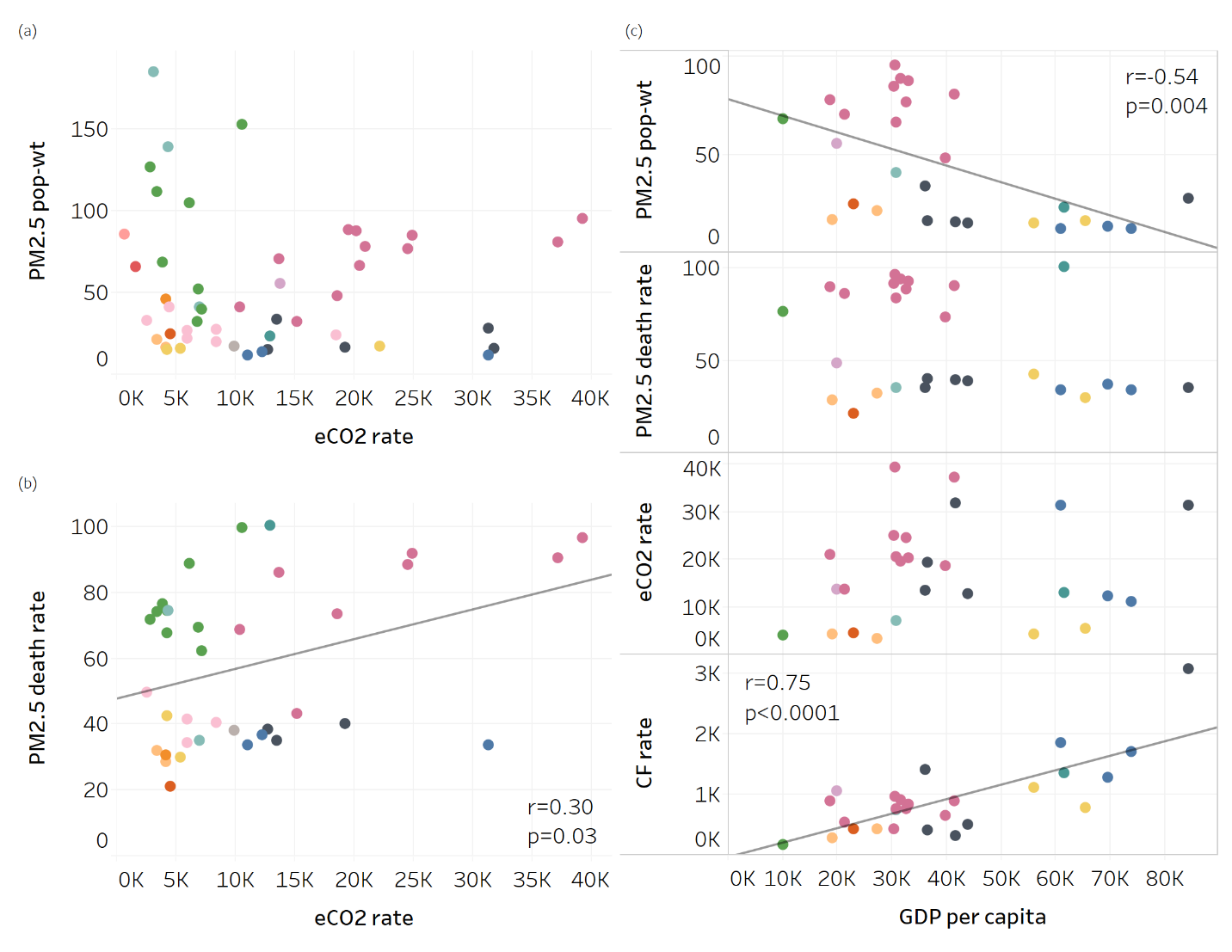


Figure S 12. As for Figure 2, but for the most populous 50 cities (n=50 for panels a and b, n=27 for panel c). See Figure 1 for color legends.

Table S 1. For each of the 250 urban areas analyzed, GBD region, population, population-weighted PM_2.5_ concentration (Popw PM_2.5_ in µg/m^3^), PM_2.5_ deaths, PM_2.5_ deaths 2.5^th^ percentile and 97.5^th^ percentile, and rank among all cities for PM_2.5_ deaths and PM_2.5_ deaths per 100,000 people. Results are all for 2016. In many cases, urban areas represent clusters of multiple cities. PM_2.5_ deaths are rounded to the nearest tens. Table is ordered by rank for PM_2.5_ deaths.

| Urban area | Country | GBD region | GBD Super-region | Pop  2016 | Popw  PM2.5 | PM2.5 deaths | PM2.5 deaths 2.5 prctl | PM2.5 deaths 97.5 prctl | Rank PM2.5 deaths | Rank PM2.5 deaths per 100,000 |
| --- | --- | --- | --- | --- | --- | --- | --- | --- | --- | --- |
| Guangzhou | China | East Asia | Southeast Asia, East Asia, and Oceania | 4.15E+07 | 48 | 30,530 | 16,840 | 45,340 | 1 | 63 |
| Cairo | Egypt | North Africa and Middle East | North Africa and Middle East | 3.42E+07 | 139 | 25,480 | 16,540 | 36,970 | 2 | 58 |
| Shanghai | China | East Asia | Southeast Asia, East Asia, and Oceania | 2.39E+07 | 76 | 21,190 | 12,760 | 30,430 | 3 | 33 |
| Kolkata | India | South Asia | South Asia | 2.31E+07 | 105 | 20,510 | 13,340 | 28,740 | 4 | 31 |
| New Delhi | India | South Asia | South Asia | 2.03E+07 | 153 | 20,240 | 13,720 | 28,190 | 5 | 5 |
| Beijing | China | East Asia | Southeast Asia, East Asia, and Oceania | 1.92E+07 | 85 | 17,660 | 10,810 | 25,230 | 6 | 19 |
| Mumbai | India | South Asia | South Asia | 1.92E+07 | 68 | 14,750 | 9,060 | 21,020 | 7 | 54 |
| Dhaka | Bangladesh | South Asia | South Asia | 1.97E+07 | 127 | 14,160 | 9,260 | 19,760 | 8 | 67 |
| Moscow | Russia | Eastern Europe | Central Europe, Eastern Europe, and Central Asia | 1.37E+07 | 23 | 13,730 | 6,200 | 22,090 | 9 | 4 |
| Tokyo | Japan | HI Asia Pacific | High-income | 3.11E+07 | 15 | 11,980 | 4,790 | 21,000 | 10 | 131 |
| Manila | Philippines | Southeast Asia | Southeast Asia, East Asia, and Oceania | 2.17E+07 | 33 | 10,800 | 5,820 | 16,020 | 11 | 102 |
| Jakarta | Indonesia | Southeast Asia | Southeast Asia, East Asia, and Oceania | 2.91E+07 | 22 | 10,060 | 4,470 | 16,200 | 12 | 154 |
| Wuxi | China | East Asia | Southeast Asia, East Asia, and Oceania | 1.10E+07 | 81 | 9,990 | 6,080 | 14,290 | 13 | 23 |
| Tianjin | China | East Asia | Southeast Asia, East Asia, and Oceania | 7.97E+06 | 95 | 7,700 | 4,820 | 10,910 | 14 | 8 |
| Chengdu | China | East Asia | Southeast Asia, East Asia, and Oceania | 8.59E+06 | 70 | 7,380 | 4,380 | 10,660 | 15 | 38 |
| Karachi | Pakistan | South Asia | South Asia | 9.74E+06 | 111 | 7,220 | 4,650 | 10,320 | 16 | 62 |
| Shantou | China | East Asia | Southeast Asia, East Asia, and Oceania | 1.03E+07 | 41 | 7,080 | 3,780 | 10,650 | 17 | 73 |
| Seoul | Korea, South | HI Asia Pacific | High-income | 1.97E+07 | 33 | 6,920 | 3,680 | 10,530 | 18 | 151 |
| Wuhan | China | East Asia | Southeast Asia, East Asia, and Oceania | 7.01E+06 | 88 | 6,590 | 4,070 | 9,370 | 19 | 16 |
| Sao Paulo | Brazil | Tropical Latin America | Latin America and Caribbean | 1.99E+07 | 21 | 6,400 | 2,980 | 10,200 | 20 | 176 |
| Hangzhou | China | East Asia | Southeast Asia, East Asia, and Oceania | 7.17E+06 | 66 | 5,980 | 3,500 | 8,690 | 21 | 43 |
| Johannesburg | South Africa | Southern sub-saharan Africa | Sub-Saharan Africa | 1.16E+07 | 55 | 5,640 | 3,580 | 7,840 | 22 | 104 |
| Nanjing | China | East Asia | Southeast Asia, East Asia, and Oceania | 6.03E+06 | 87 | 5,590 | 3,430 | 7,970 | 23 | 18 |
| Bangkok | Thailand | Southeast Asia | Southeast Asia, East Asia, and Oceania | 1.32E+07 | 28 | 5,310 | 2,780 | 8,080 | 24 | 121 |
| Chennai | India | South Asia | South Asia | 8.48E+06 | 40 | 5,290 | 2,940 | 7,760 | 25 | 84 |
| Hyderabad | India | South Asia | South Asia | 7.48E+06 | 52 | 5,190 | 3,050 | 7,490 | 26 | 72 |
| Shenyeng | China | East Asia | Southeast Asia, East Asia, and Oceania | 5.39E+06 | 94 | 5,180 | 3,230 | 7,350 | 27 | 10 |
| Los Angeles | United States | HI North America | High-income | 1.41E+07 | 13 | 5,170 | 2,060 | 8,960 | 28 | 145 |
| New York | United States | HI North America | High-income | 1.51E+07 | 12 | 5,120 | 1,920 | 9,100 | 29 | 158 |
| Xian | China | East Asia | Southeast Asia, East Asia, and Oceania | 5.47E+06 | 78 | 4,920 | 2,980 | 7,050 | 30 | 26 |
| Osaka | Japan | HI Asia Pacific | High-income | 1.22E+07 | 16 | 4,920 | 2,040 | 8,470 | 30 | 122 |
| Sohag | Egypt | North Africa and Middle East | North Africa and Middle East | 5.60E+06 | 185 | 4,530 | 3,040 | 6,530 | 32 | 49 |
| Buenos Aires | Argentina | HI Southern Latin America | High-income | 1.17E+07 | 17 | 4,490 | 1,910 | 7,500 | 33 | 136 |
| Istanbul | Turkey | North Africa and Middle East | North Africa and Middle East | 1.26E+07 | 41 | 4,430 | 2,440 | 6,600 | 34 | 149 |
| Ho Chi Minh City | Vietnam | Southeast Asia | Southeast Asia, East Asia, and Oceania | 1.02E+07 | 26 | 4,230 | 2,020 | 6,740 | 35 | 120 |
| Mexico City | Mexico | Central Latin America | Latin America and Caribbean | 1.99E+07 | 24 | 4,180 | 2,040 | 6,490 | 36 | 229 |
| London | United Kingdom | HI Western Europe | High-income | 9.65E+06 | 15 | 4,100 | 1,670 | 7,070 | 37 | 115 |
| Chongqing | China | East Asia | Southeast Asia, East Asia, and Oceania | 4.72E+06 | 67 | 4,010 | 2,370 | 5,800 | 38 | 40 |
| Wenzhou | China | East Asia | Southeast Asia, East Asia, and Oceania | 5.40E+06 | 45 | 3,870 | 2,110 | 5,780 | 39 | 68 |
| Zhengzhou | China | East Asia | Southeast Asia, East Asia, and Oceania | 4.17E+06 | 84 | 3,820 | 2,330 | 5,450 | 40 | 20 |
| Bangalore | India | South Asia | South Asia | 6.51E+06 | 32 | 3,690 | 1,950 | 5,530 | 41 | 91 |
| Harbin | China | East Asia | Southeast Asia, East Asia, and Oceania | 4.02E+06 | 80 | 3,610 | 2,190 | 5,170 | 42 | 27 |
| Surat | India | South Asia | South Asia | 4.44E+06 | 73 | 3,480 | 2,170 | 4,950 | 43 | 51 |
| Pune | India | South Asia | South Asia | 4.64E+06 | 62 | 3,450 | 2,090 | 4,930 | 44 | 61 |
| Alexandria | Egypt | North Africa and Middle East | North Africa and Middle East | 4.76E+06 | 118 | 3,410 | 2,170 | 4,950 | 45 | 69 |
| Hong Kong | China | East Asia | Southeast Asia, East Asia, and Oceania | 5.08E+06 | 34 | 3,280 | 1,700 | 5,010 | 46 | 82 |
| Lahore | Pakistan | South Asia | South Asia | 4.79E+06 | 86 | 3,240 | 2,010 | 4,660 | 47 | 77 |
| Shijianzhuang | China | East Asia | Southeast Asia, East Asia, and Oceania | 3.20E+06 | 110 | 3,230 | 2,060 | 4,560 | 48 | 3 |
| Taipei | Taiwan | East Asia | Southeast Asia, East Asia, and Oceania | 7.38E+06 | 32 | 3,190 | 1,740 | 4,770 | 49 | 114 |
| Lagos | Nigeria | Western sub-saharan Africa | Sub-Saharan Africa | 1.01E+07 | 66 | 3,120 | 2,220 | 4,050 | 50 | 185 |
| Changchun | China | East Asia | Southeast Asia, East Asia, and Oceania | 3.26E+06 | 86 | 3,040 | 1,870 | 4,330 | 51 | 17 |
| St. Petersburg | Russia | Eastern Europe | Central Europe, Eastern Europe, and Central Asia | 4.18E+06 | 11 | 3,010 | 1,050 | 5,510 | 52 | 66 |
| Paris | French Republic | HI Western Europe | High-income | 9.88E+06 | 16 | 2,950 | 1,260 | 5,010 | 53 | 192 |
| Hanoi | Vietnam | Southeast Asia | Southeast Asia, East Asia, and Oceania | 5.69E+06 | 41 | 2,890 | 1,550 | 4,340 | 54 | 45 |
| Qingdao | China | East Asia | Southeast Asia, East Asia, and Oceania | 3.52E+06 | 62 | 2,890 | 1,680 | 4,210 | 54 | 97 |
| Cologne | Germany | HI Western Europe | High-income | 5.75E+06 | 17 | 2,850 | 1,250 | 4,730 | 56 | 103 |
| Taiyuan | China | East Asia | Southeast Asia, East Asia, and Oceania | 3.28E+06 | 71 | 2,840 | 1,700 | 4,100 | 57 | 36 |
| Rio de Janeiro | Brazil | Tropical Latin America | Latin America and Caribbean | 9.82E+06 | 17 | 2,810 | 1,190 | 4,670 | 58 | 204 |
| Kinshasa | Congo (Kinshasa) | Central sub-saharan Africa | Sub-Saharan Africa | 6.10E+06 | 86 | 2,800 | 1,870 | 3,790 | 59 | 79 |
| Quanzhou | China | East Asia | Southeast Asia, East Asia, and Oceania | 4.24E+06 | 38 | 2,800 | 1,470 | 4,260 | 59 | 112 |
| Dalian | China | East Asia | Southeast Asia, East Asia, and Oceania | 3.17E+06 | 65 | 2,650 | 1,550 | 3,850 | 61 | 42 |
| Jinan | China | East Asia | Southeast Asia, East Asia, and Oceania | 3.07E+06 | 69 | 2,630 | 1,560 | 3,790 | 62 | 39 |
| Chicago | United States | HI North America | High-income | 7.73E+06 | 12 | 2,610 | 980 | 4,640 | 63 | 161 |
| Nagoya | Japan | HI Asia Pacific | High-income | 6.48E+06 | 15 | 2,550 | 1,040 | 4,430 | 64 | 126 |
| Asyut | Egypt | North Africa and Middle East | North Africa and Middle East | 3.08E+06 | 190 | 2,510 | 1,690 | 3,620 | 65 | 46 |
| Lima | Peru | Andean Latin America | Latin America and Caribbean | 7.94E+06 | 46 | 2,450 | 1,570 | 3,380 | 66 | 186 |
| Kathmandu | Nepal | South Asia | South Asia | 3.47E+06 | 77 | 2,350 | 1,480 | 3,320 | 67 | 75 |
| Nanchang | China | East Asia | Southeast Asia, East Asia, and Oceania | 2.95E+06 | 56 | 2,330 | 1,330 | 3,420 | 68 | 50 |
| Asansol | India | South Asia | South Asia | 2.62E+06 | 104 | 2,320 | 1,510 | 3,250 | 69 | 34 |
| Rangoon | Myanmar | Southeast Asia | Southeast Asia, East Asia, and Oceania | 4.79E+06 | 57 | 2,300 | 1,370 | 3,290 | 70 | 107 |
| Singapore | Singapore | HI Asia Pacific | High-income | 6.48E+06 | 28 | 2,290 | 1,200 | 3,460 | 71 | 148 |
| Peshawar | Pakistan | South Asia | South Asia | 3.66E+06 | 66 | 2,260 | 1,350 | 3,290 | 72 | 85 |
| Kuala Lumpur | Malaysia | Southeast Asia | Southeast Asia, East Asia, and Oceania | 5.48E+06 | 24 | 2,120 | 1,030 | 3,290 | 73 | 130 |
| Surabaya | Indonesia | Southeast Asia | Southeast Asia, East Asia, and Oceania | 6.10E+06 | 20 | 2,020 | 870 | 3,300 | 74 | 165 |
| Faisalabad | Pakistan | South Asia | South Asia | 2.94E+06 | 88 | 2,000 | 1,250 | 2,870 | 75 | 74 |
| Fuzhou | China | East Asia | Southeast Asia, East Asia, and Oceania | 3.05E+06 | 35 | 2,000 | 1,040 | 3,040 | 75 | 81 |
| Milan | Italy | HI Western Europe | High-income | 3.96E+06 | 24 | 1,980 | 960 | 3,150 | 77 | 101 |
| Kunming | China | East Asia | Southeast Asia, East Asia, and Oceania | 2.94E+06 | 36 | 1,930 | 1,010 | 2,940 | 78 | 80 |
| Kiev | Ukraine | Eastern Europe | Central Europe, Eastern Europe, and Central Asia | 1.54E+06 | 25 | 1,920 | 880 | 3,040 | 79 | 1 |
| Ningbo | China | East Asia | Southeast Asia, East Asia, and Oceania | 2.47E+06 | 54 | 1,890 | 1,060 | 2,790 | 80 | 53 |
| Chittagong | Bangladesh | South Asia | South Asia | 3.13E+06 | 76 | 1,880 | 1,140 | 2,670 | 81 | 87 |
| Bandung | Indonesia | Southeast Asia | Southeast Asia, East Asia, and Oceania | 5.07E+06 | 21 | 1,720 | 750 | 2,780 | 82 | 159 |
| Tehran | Iran | North Africa and Middle East | North Africa and Middle East | 5.15E+06 | 41 | 1,700 | 920 | 2,550 | 83 | 167 |
| Athens | Greece | HI Western Europe | High-income | 3.03E+06 | 13 | 1,690 | 650 | 3,030 | 84 | 94 |
| Kano | Nigeria | Western sub-saharan Africa | Sub-Saharan Africa | 3.36E+06 | 365 | 1,680 | 1,290 | 2,050 | 85 | 100 |
| Bucharest | Romania | Central Europe | Central Europe, Eastern Europe, and Central Asia | 1.71E+06 | 25 | 1,650 | 770 | 2,640 | 86 | 9 |
| Colombo | Sri Lanka | Southeast Asia | Southeast Asia, East Asia, and Oceania | 4.43E+06 | 32 | 1,640 | 830 | 2,510 | 87 | 142 |
| Budapest | Hungary | Central Europe | Central Europe, Eastern Europe, and Central Asia | 1.67E+06 | 29 | 1,600 | 800 | 2,480 | 88 | 12 |
| Baghdad | Iraq | North Africa and Middle East | North Africa and Middle East | 3.31E+06 | 91 | 1,580 | 960 | 2,300 | 89 | 109 |
| Baotou | China | East Asia | Southeast Asia, East Asia, and Oceania | 1.92E+06 | 59 | 1,560 | 900 | 2,280 | 90 | 47 |
| Naples | Italy | HI Western Europe | High-income | 3.62E+06 | 17 | 1,530 | 670 | 2,570 | 91 | 117 |
| San Francisco | United States | HI North America | High-income | 4.55E+06 | 11 | 1,500 | 550 | 2,700 | 92 | 168 |
| Dallas | United States | HI North America | High-income | 4.94E+06 | 10 | 1,490 | 510 | 2,760 | 93 | 191 |
| Manchester | United Kingdom | HI Western Europe | High-income | 3.79E+06 | 13 | 1,480 | 570 | 2,640 | 94 | 128 |
| Tangshan | China | East Asia | Southeast Asia, East Asia, and Oceania | 1.63E+06 | 80 | 1,460 | 880 | 2,090 | 95 | 28 |
| Dar es Salaam | United Republic of Tanzania | Eastern sub-saharan Africa | Sub-Saharan Africa | 4.90E+06 | 30 | 1,420 | 770 | 2,100 | 96 | 199 |
| Santiago | Chile | HI Southern Latin America | High-income | 4.34E+06 | 32 | 1,400 | 740 | 2,110 | 97 | 174 |
| Surakarta | Indonesia | Southeast Asia | Southeast Asia, East Asia, and Oceania | 4.29E+06 | 19 | 1,380 | 580 | 2,280 | 98 | 175 |
| Anshan | China | East Asia | Southeast Asia, East Asia, and Oceania | 1.45E+06 | 90 | 1,370 | 850 | 1,950 | 99 | 14 |
| Casablanca | Morocco | North Africa and Middle East | North Africa and Middle East | 3.86E+06 | 24 | 1,360 | 640 | 2,120 | 100 | 150 |
| Miami | United States | HI North America | High-income | 5.45E+06 | 8 | 1,350 | 380 | 2,690 | 101 | 218 |
| Jiaojing | China | East Asia | Southeast Asia, East Asia, and Oceania | 1.88E+06 | 45 | 1,350 | 730 | 2,010 | 101 | 70 |
| Houston | United States | HI North America | High-income | 4.70E+06 | 9 | 1,340 | 440 | 2,540 | 103 | 203 |
| Accra | Ghana | Western sub-saharan Africa | Sub-Saharan Africa | 3.43E+06 | 49 | 1,320 | 810 | 1,850 | 104 | 134 |
| Algiers | Algeria | North Africa and Middle East | North Africa and Middle East | 4.30E+06 | 38 | 1,310 | 680 | 1,990 | 105 | 55 |
| Nagpur | India | South Asia | South Asia | 1.72E+06 | 68 | 1,310 | 800 | 1,870 | 105 | 189 |
| Philadelphia | United States | HI North America | High-income | 3.83E+06 | 12 | 1,300 | 490 | 2,310 | 107 | 155 |
| Toronto | Canada | HI North America | High-income | 5.32E+06 | 10 | 1,240 | 410 | 2,340 | 108 | 44 |
| Luoyang | China | East Asia | Southeast Asia, East Asia, and Oceania | 1.49E+06 | 64 | 1,240 | 730 | 1,800 | 108 | 223 |
| Luanda | Angola | Central sub-saharan Africa | Sub-Saharan Africa | 4.25E+06 | 49 | 1,220 | 750 | 1,720 | 110 | 200 |
| Berlin | Germany | HI Western Europe | High-income | 2.49E+06 | 16 | 1,200 | 510 | 2,010 | 111 | 60 |
| Warsaw | Poland | Central Europe | Central Europe, Eastern Europe, and Central Asia | 1.62E+06 | 32 | 1,200 | 630 | 1,830 | 111 | 106 |
| Tashkent | Uzbekistan | Central Asia | Central Europe, Eastern Europe, and Central Asia | 1.64E+06 | 70 | 1,190 | 690 | 1,760 | 113 | 35 |
| Linyi | China | East Asia | Southeast Asia, East Asia, and Oceania | 1.36E+06 | 75 | 1,190 | 720 | 1,720 | 113 | 65 |
| Zibo | China | East Asia | Southeast Asia, East Asia, and Oceania | 1.36E+06 | 73 | 1,180 | 700 | 1,700 | 115 | 37 |
| Washington, D.C. | United States | HI North America | High-income | 3.51E+06 | 11 | 1,170 | 430 | 2,090 | 116 | 164 |
| Dhanbad | India | South Asia | South Asia | 1.31E+06 | 103 | 1,160 | 760 | 1,630 | 117 | 32 |
| Lucknow | India | South Asia | South Asia | 1.15E+06 | 141 | 1,120 | 750 | 1,560 | 118 | 6 |
| Port-au-Prince | Haiti | Caribbean | Latin America and Caribbean | 2.36E+06 | 31 | 1,110 | 540 | 1,710 | 119 | 110 |
| Rome | Italy | HI Western Europe | High-income | 2.73E+06 | 15 | 1,070 | 450 | 1,840 | 120 | 127 |
| Handan | China | East Asia | Southeast Asia, East Asia, and Oceania | 1.12E+06 | 94 | 1,070 | 660 | 1,520 | 120 | 13 |
| Ouagadougou | Burkina Faso | Western sub-saharan Africa | Sub-Saharan Africa | 1.88E+06 | 131 | 1,050 | 760 | 1,340 | 122 | 113 |
| Chandigarh | India | South Asia | South Asia | 1.18E+06 | 107 | 1,050 | 680 | 1,470 | 122 | 29 |
| Cebu | Philippines | Southeast Asia | Southeast Asia, East Asia, and Oceania | 2.42E+06 | 23 | 1,050 | 510 | 1,630 | 122 | 95 |
| San Diego | United States | HI North America | High-income | 3.73E+06 | 12 | 1,040 | 380 | 1,880 | 125 | 210 |
| Detroit | United States | HI North America | High-income | 3.08E+06 | 12 | 1,040 | 400 | 1,830 | 125 | 202 |
| Orlu | Nigeria | Western sub-saharan Africa | Sub-Saharan Africa | 3.64E+06 | 56 | 1,040 | 730 | 1,370 | 125 | 162 |
| Barcelona | Kingdom of Spain | HI Western Europe | High-income | 3.41E+06 | 12 | 1,030 | 390 | 1,840 | 128 | 190 |
| Madrid | Kingdom of Spain | HI Western Europe | High-income | 3.62E+06 | 11 | 1,020 | 370 | 1,870 | 129 | 209 |
| Phoenix | United States | HI North America | High-income | 3.49E+06 | 10 | 1,010 | 340 | 1,900 | 130 | 198 |
| Taichung | Taiwan | East Asia | Southeast Asia, East Asia, and Oceania | 2.35E+06 | 32 | 990 | 530 | 1,490 | 131 | 118 |
| Medan | Indonesia | Southeast Asia | Southeast Asia, East Asia, and Oceania | 2.64E+06 | 26 | 980 | 460 | 1,550 | 132 | 141 |
| Santo Domingo | Dominican Republic | Caribbean | Latin America and Caribbean | 3.10E+06 | 35 | 960 | 480 | 1,490 | 133 | 184 |
| Belo Horizonte | Brazil | Tropical Latin America | Latin America and Caribbean | 4.11E+06 | 12 | 960 | 340 | 1,720 | 133 | 21 |
| Kochi | India | South Asia | South Asia | 1.69E+06 | 32 | 960 | 500 | 1,430 | 133 | 93 |
| Anyang | China | East Asia | Southeast Asia, East Asia, and Oceania | 1.05E+06 | 81 | 960 | 580 | 1,370 | 133 | 224 |
| Birmingham | United Kingdom | HI Western Europe | High-income | 2.47E+06 | 13 | 950 | 360 | 1,700 | 137 | 135 |
| Rotterdam | Kingdom of the Netherlands | HI Western Europe | High-income | 2.41E+06 | 17 | 920 | 400 | 1,550 | 138 | 137 |
| Katowice | Poland | Central Europe | Central Europe, Eastern Europe, and Central Asia | 1.19E+06 | 35 | 920 | 490 | 1,390 | 138 | 52 |
| Busan | Korea, South | HI Asia Pacific | High-income | 2.89E+06 | 26 | 900 | 450 | 1,420 | 140 | 182 |
| Guadalajara | Mexico | Central Latin America | Latin America and Caribbean | 4.59E+06 | 20 | 870 | 400 | 1,400 | 141 | 238 |
| Monterrey | Mexico | Central Latin America | Latin America and Caribbean | 4.23E+06 | 23 | 860 | 410 | 1,350 | 142 | 172 |
| Semarang | Indonesia | Southeast Asia | Southeast Asia, East Asia, and Oceania | 2.65E+06 | 19 | 860 | 370 | 1,420 | 142 | 232 |
| Agra | India | South Asia | South Asia | 8.91E+05 | 130 | 850 | 570 | 1,190 | 144 | 11 |
| Bamako | Mali | Western sub-saharan Africa | Sub-Saharan Africa | 2.51E+06 | 85 | 830 | 550 | 1,120 | 145 | 170 |
| Xining | China | East Asia | Southeast Asia, East Asia, and Oceania | 9.67E+05 | 59 | 780 | 450 | 1,140 | 146 | 48 |
| Vishakhapatnam | India | South Asia | South Asia | 1.14E+06 | 48 | 770 | 450 | 1,120 | 147 | 76 |
| Curitiba | Brazil | Tropical Latin America | Latin America and Caribbean | 2.67E+06 | 17 | 760 | 320 | 1,260 | 148 | 208 |
| Almaty | Kazakhstan | Central Asia | Central Europe, Eastern Europe, and Central Asia | 1.25E+06 | 28 | 750 | 350 | 1,180 | 149 | 88 |
| Ankara | Turkey | North Africa and Middle East | North Africa and Middle East | 1.93E+06 | 48 | 720 | 410 | 1,060 | 150 | 140 |
| Montreal | Canada | HI North America | High-income | 3.00E+06 | 10 | 710 | 240 | 1,340 | 151 | 221 |
| Chelyabinsk | Russia | Eastern Europe | Central Europe, Eastern Europe, and Central Asia | 7.30E+05 | 21 | 710 | 310 | 1,150 | 151 | 7 |
| Yantai | China | East Asia | Southeast Asia, East Asia, and Oceania | 9.43E+05 | 49 | 700 | 390 | 1,040 | 153 | 59 |
| Indore | India | South Asia | South Asia | 9.03E+05 | 66 | 680 | 420 | 970 | 154 | 56 |
| Mandalay | Myanmar | Southeast Asia | Southeast Asia, East Asia, and Oceania | 1.31E+06 | 63 | 660 | 400 | 930 | 155 | 99 |
| Zhenjiang | China | East Asia | Southeast Asia, East Asia, and Oceania | 7.23E+05 | 80 | 660 | 400 | 940 | 155 | 22 |
| Ibadan | Nigeria | Western sub-saharan Africa | Sub-Saharan Africa | 2.01E+06 | 73 | 650 | 470 | 840 | 157 | 173 |
| Kollam | India | South Asia | South Asia | 1.24E+06 | 27 | 650 | 320 | 990 | 157 | 96 |
| Phnom Penh | Cambodia | Southeast Asia | Southeast Asia, East Asia, and Oceania | 1.68E+06 | 34 | 650 | 330 | 980 | 157 | 132 |
| Lisbon | Portugal | HI Western Europe | High-income | 1.73E+06 | 11 | 640 | 210 | 1,230 | 160 | 144 |
| Seattle | United States | HI North America | High-income | 2.47E+06 | 8 | 620 | 180 | 1,230 | 161 | 216 |
| Boston | United States | HI North America | High-income | 2.12E+06 | 10 | 620 | 210 | 1,160 | 161 | 116 |
| Leeds | United Kingdom | HI Western Europe | High-income | 1.61E+06 | 13 | 620 | 240 | 1,110 | 161 | 196 |
| Vienna | Austria | HI Western Europe | High-income | 1.46E+06 | 20 | 620 | 290 | 990 | 161 | 133 |
| Turin | Italy | HI Western Europe | High-income | 1.27E+06 | 22 | 610 | 290 | 970 | 165 | 41 |
| Bhilai | India | South Asia | South Asia | 7.21E+05 | 96 | 610 | 390 | 860 | 165 | 108 |
| Las Vegas | United States | HI North America | High-income | 2.05E+06 | 10 | 600 | 200 | 1,130 | 167 | 195 |
| Bogota | Colombia | Central Latin America | Latin America and Caribbean | 2.81E+06 | 20 | 590 | 270 | 940 | 168 | 230 |
| Sydney | Australia | HI Australasia | High-income | 3.84E+06 | 7 | 590 | 140 | 1,260 | 168 | 247 |
| Guatemala | Guatemala | Central Latin America | Latin America and Caribbean | 2.24E+06 | 32 | 580 | 340 | 840 | 170 | 214 |
| Marietta | United States | HI North America | High-income | 1.95E+06 | 10 | 580 | 200 | 1,080 | 170 | 194 |
| Fukuoka | Japan | HI Asia Pacific | High-income | 1.37E+06 | 17 | 580 | 250 | 980 | 170 | 119 |
| Salvador | Brazil | Tropical Latin America | Latin America and Caribbean | 3.03E+06 | 9 | 560 | 160 | 1,100 | 173 | 240 |
| Campinas | Brazil | Tropical Latin America | Latin America and Caribbean | 1.74E+06 | 20 | 550 | 250 | 880 | 174 | 86 |
| Prague | Czech Republic | Central Europe | Central Europe, Eastern Europe, and Central Asia | 9.12E+05 | 19 | 550 | 250 | 890 | 174 | 2 |
| Zaporizhzhya | Ukraine | Eastern Europe | Central Europe, Eastern Europe, and Central Asia | 4.58E+05 | 23 | 550 | 250 | 880 | 174 | 83 |
| Zhanjiang | China | East Asia | Southeast Asia, East Asia, and Oceania | 8.67E+05 | 32 | 550 | 280 | 840 | 174 | 181 |
| Luohe | China | East Asia | Southeast Asia, East Asia, and Oceania | 5.94E+05 | 80 | 540 | 330 | 770 | 178 | 25 |
| Melbourne | Australia | HI Australasia | High-income | 3.40E+06 | 7 | 530 | 130 | 1,130 | 179 | 246 |
| Caracas | Venezuela | Central Latin America | Latin America and Caribbean | 1.82E+06 | 31 | 520 | 270 | 790 | 180 | 249 |
| Fortaleza | Brazil | Tropical Latin America | Latin America and Caribbean | 3.13E+06 | 8 | 520 | 130 | 1,070 | 180 | 64 |
| Belgrade | Republic of Serbia | Central Europe | Central Europe, Eastern Europe, and Central Asia | 7.18E+05 | 23 | 520 | 240 | 850 | 180 | 245 |
| Amman | Jordan | North Africa and Middle East | North Africa and Middle East | 2.40E+06 | 42 | 520 | 280 | 770 | 180 | 205 |
| Nairobi | Kenya | Eastern sub-saharan Africa | Sub-Saharan Africa | 3.99E+06 | 15 | 520 | 200 | 910 | 180 | 227 |
| Denver | United States | HI North America | High-income | 1.97E+06 | 8 | 510 | 150 | 1,010 | 185 | 213 |
| Angeles | Philippines | Southeast Asia | Southeast Asia, East Asia, and Oceania | 1.09E+06 | 28 | 510 | 260 | 770 | 185 | 111 |
| Xuchang | China | East Asia | Southeast Asia, East Asia, and Oceania | 5.57E+05 | 81 | 500 | 310 | 720 | 187 | 24 |
| Niteroi | Brazil | Tropical Latin America | Latin America and Caribbean | 1.62E+06 | 18 | 480 | 210 | 790 | 188 | 178 |
| Harare | Zimbabwe | Southern sub-saharan Africa | Sub-Saharan Africa | 1.52E+06 | 36 | 480 | 270 | 710 | 188 | 193 |
| Rabat | Morocco | North Africa and Middle East | North Africa and Middle East | 1.38E+06 | 22 | 470 | 220 | 740 | 190 | 98 |
| Haiphong | Vietnam | Southeast Asia | Southeast Asia, East Asia, and Oceania | 9.33E+05 | 41 | 470 | 250 | 710 | 190 | 156 |
| Goiania | Brazil | Tropical Latin America | Latin America and Caribbean | 1.87E+06 | 13 | 460 | 170 | 810 | 192 | 219 |
| Minneapolis | United States | HI North America | High-income | 1.57E+06 | 9 | 450 | 150 | 840 | 193 | 206 |
| Denpasar | Indonesia | Southeast Asia | Southeast Asia, East Asia, and Oceania | 1.45E+06 | 17 | 450 | 180 | 750 | 193 | 187 |
| Baltimore | United States | HI North America | High-income | 1.34E+06 | 11 | 440 | 160 | 800 | 195 | 169 |
| Port Harcourt | Nigeria | Western sub-saharan Africa | Sub-Saharan Africa | 1.72E+06 | 44 | 440 | 290 | 590 | 195 | 215 |
| Puebla | Mexico | Central Latin America | Latin America and Caribbean | 2.07E+06 | 23 | 420 | 200 | 660 | 197 | 233 |
| Ciudad Juarez | Mexico | Central Latin America | Latin America and Caribbean | 1.85E+06 | 18 | 410 | 170 | 700 | 198 | 212 |
| Orlando | United States | HI North America | High-income | 1.53E+06 | 9 | 410 | 130 | 800 | 198 | 105 |
| Stuttgart | Germany | HI Western Europe | High-income | 8.51E+05 | 16 | 410 | 180 | 690 | 198 | 226 |
| Meerut | India | South Asia | South Asia | 4.34E+05 | 128 | 410 | 270 | 570 | 198 | 15 |
| Rawalpindi | Pakistan | South Asia | South Asia | 6.78E+05 | 59 | 400 | 240 | 590 | 202 | 89 |
| Coimbatore | India | South Asia | South Asia | 6.79E+05 | 36 | 400 | 220 | 600 | 202 | 90 |
| Maracaibo | Venezuela | Central Latin America | Latin America and Caribbean | 1.25E+06 | 40 | 390 | 210 | 580 | 204 | 180 |
| Cirebon | Indonesia | Southeast Asia | Southeast Asia, East Asia, and Oceania | 1.14E+06 | 21 | 390 | 170 | 630 | 204 | 157 |
| Columbus | United States | HI North America | High-income | 1.07E+06 | 12 | 380 | 140 | 660 | 206 | 124 |
| Glasgow | United Kingdom | HI Western Europe | High-income | 9.60E+05 | 14 | 380 | 150 | 670 | 206 | 146 |
| Aleppo | Syria | North Africa and Middle East | North Africa and Middle East | 1.04E+06 | 45 | 380 | 200 | 570 | 206 | 153 |
| Porto | Portugal | HI Western Europe | High-income | 1.03E+06 | 11 | 370 | 120 | 720 | 209 | 30 |
| Ludhiana | India | South Asia | South Asia | 4.19E+05 | 107 | 370 | 240 | 520 | 209 | 147 |
| San Antonio | United States | HI North America | High-income | 1.34E+06 | 9 | 360 | 110 | 700 | 211 | 211 |
| Marrakesh | Morocco | North Africa and Middle East | North Africa and Middle East | 9.12E+05 | 30 | 360 | 180 | 540 | 211 | 125 |
| Fez | Morocco | North Africa and Middle East | North Africa and Middle East | 9.71E+05 | 26 | 360 | 180 | 550 | 211 | 143 |
| Tegal | Indonesia | Southeast Asia | Southeast Asia, East Asia, and Oceania | 1.08E+06 | 20 | 360 | 150 | 580 | 211 | 166 |
| Barranquilla | Colombia | Central Latin America | Latin America and Caribbean | 1.43E+06 | 26 | 340 | 170 | 530 | 215 | 220 |
| Douala | Cameroon | Western sub-saharan Africa | Sub-Saharan Africa | 5.10E+05 | 137 | 340 | 240 | 440 | 215 | 78 |
| Cleveland | United States | HI North America | High-income | 1.05E+06 | 11 | 330 | 120 | 610 | 217 | 179 |
| Vitiria | Brazil | Tropical Latin America | Latin America and Caribbean | 1.47E+06 | 12 | 330 | 120 | 600 | 217 | 225 |
| Ashgabat | Turkmenistan | Central Asia | Central Europe, Eastern Europe, and Central Asia | 5.82E+05 | 41 | 330 | 170 | 500 | 217 | 92 |
| San Jose | Costa Rica | Central Latin America | Latin America and Caribbean | 1.66E+06 | 22 | 310 | 150 | 490 | 220 | 57 |
| Kansas City | United States | HI North America | High-income | 1.09E+06 | 9 | 310 | 100 | 590 | 220 | 239 |
| Cuttack | India | South Asia | South Asia | 4.17E+05 | 64 | 310 | 190 | 450 | 220 | 201 |
| Gaza | Israel | HI Western Europe | High-income | 1.25E+06 | 23 | 300 | 140 | 470 | 223 | 222 |
| Maebashi | Japan | HI Asia Pacific | High-income | 7.98E+05 | 14 | 300 | 120 | 530 | 223 | 139 |
| Leon | Mexico | Central Latin America | Latin America and Caribbean | 1.48E+06 | 21 | 290 | 140 | 460 | 225 | 236 |
| Valencia | Kingdom of Spain | HI Western Europe | High-income | 1.00E+06 | 11 | 290 | 100 | 520 | 225 | 123 |
| Riyadh | Saudi Arabia | North Africa and Middle East | North Africa and Middle East | 7.21E+05 | 280 | 290 | 210 | 400 | 225 | 207 |
| Dublin | Ireland | HI Western Europe | High-income | 1.09E+06 | 11 | 270 | 100 | 500 | 228 | 217 |
| Cordoba | Argentina | HI Southern Latin America | High-income | 7.35E+05 | 14 | 250 | 100 | 430 | 229 | 129 |
| Sdo Lu1s | Brazil | Tropical Latin America | Latin America and Caribbean | 1.23E+06 | 10 | 250 | 80 | 470 | 229 | 160 |
| Kitakyushu | Japan | HI Asia Pacific | High-income | 6.43E+05 | 15 | 250 | 100 | 440 | 229 | 234 |
| Calgary | Canada | HI North America | High-income | 1.15E+06 | 8 | 240 | 70 | 470 | 232 | 188 |
| Virginia Beach | United States | HI North America | High-income | 7.96E+05 | 10 | 240 | 80 | 450 | 232 | 152 |
| Gwangju | Korea, South | HI Asia Pacific | High-income | 6.88E+05 | 33 | 240 | 130 | 370 | 232 | 231 |
| Tasikmalaya | Indonesia | Southeast Asia | Southeast Asia, East Asia, and Oceania | 6.86E+05 | 17 | 210 | 90 | 360 | 235 | 183 |
| Torreon | Mexico | Central Latin America | Latin America and Caribbean | 1.02E+06 | 20 | 200 | 90 | 310 | 236 | 177 |
| Bursa | Turkey | North Africa and Middle East | North Africa and Middle East | 6.22E+05 | 34 | 200 | 100 | 300 | 236 | 237 |
| San Luis Potosi | Mexico | Central Latin America | Latin America and Caribbean | 1.06E+06 | 18 | 190 | 80 | 310 | 238 | 163 |
| Bandar Lampung | Indonesia | Southeast Asia | Southeast Asia, East Asia, and Oceania | 5.72E+05 | 20 | 190 | 80 | 310 | 238 | 241 |
| Pakalongan | Indonesia | Southeast Asia | Southeast Asia, East Asia, and Oceania | 5.36E+05 | 19 | 180 | 70 | 290 | 240 | 171 |
| Merida | Mexico | Central Latin America | Latin America and Caribbean | 9.50E+05 | 16 | 160 | 70 | 270 | 241 | 244 |
| Tampico | Mexico | Central Latin America | Latin America and Caribbean | 7.62E+05 | 22 | 150 | 70 | 240 | 242 | 235 |
| Stockholm | Sweden | HI Western Europe | High-income | 1.16E+06 | 5 | 150 | - | 460 | 242 | 250 |
| Saltillo | Mexico | Central Latin America | Latin America and Caribbean | 7.71E+05 | 17 | 140 | 60 | 220 | 244 | 243 |
| Acapulco | Mexico | Central Latin America | Latin America and Caribbean | 7.15E+05 | 17 | 130 | 50 | 210 | 245 | 242 |
| Tuxtla Gutierrez | Mexico | Central Latin America | Latin America and Caribbean | 5.76E+05 | 26 | 120 | 60 | 190 | 246 | 228 |
| Charlotte | United States | HI North America | High-income | 3.91E+05 | 10 | 110 | 40 | 210 | 247 | 197 |
| Haarlem | Kingdom of the Netherlands | HI Western Europe | High-income | 2.84E+05 | 16 | 110 | 50 | 180 | 247 | 138 |
| Cancun | Mexico | Central Latin America | Latin America and Caribbean | 6.89E+05 | 13 | 100 | 40 | 180 | 249 | 248 |
| Shibin el Kom | Egypt | North Africa and Middle East | North Africa and Middle East | 4.38E+04 | 113 | 30 | 20 | 50 | 250 | 71 |
